# Supplementary figures and images for: Identification and validation of immune related core transcription factors GTF2I in NAFLD
Source: PeerJ. 2022 Jul 21;10:e13735. doi: 10.7717/peerj.13735 (PMC9308966; doi:10.7717/peerj.13735)

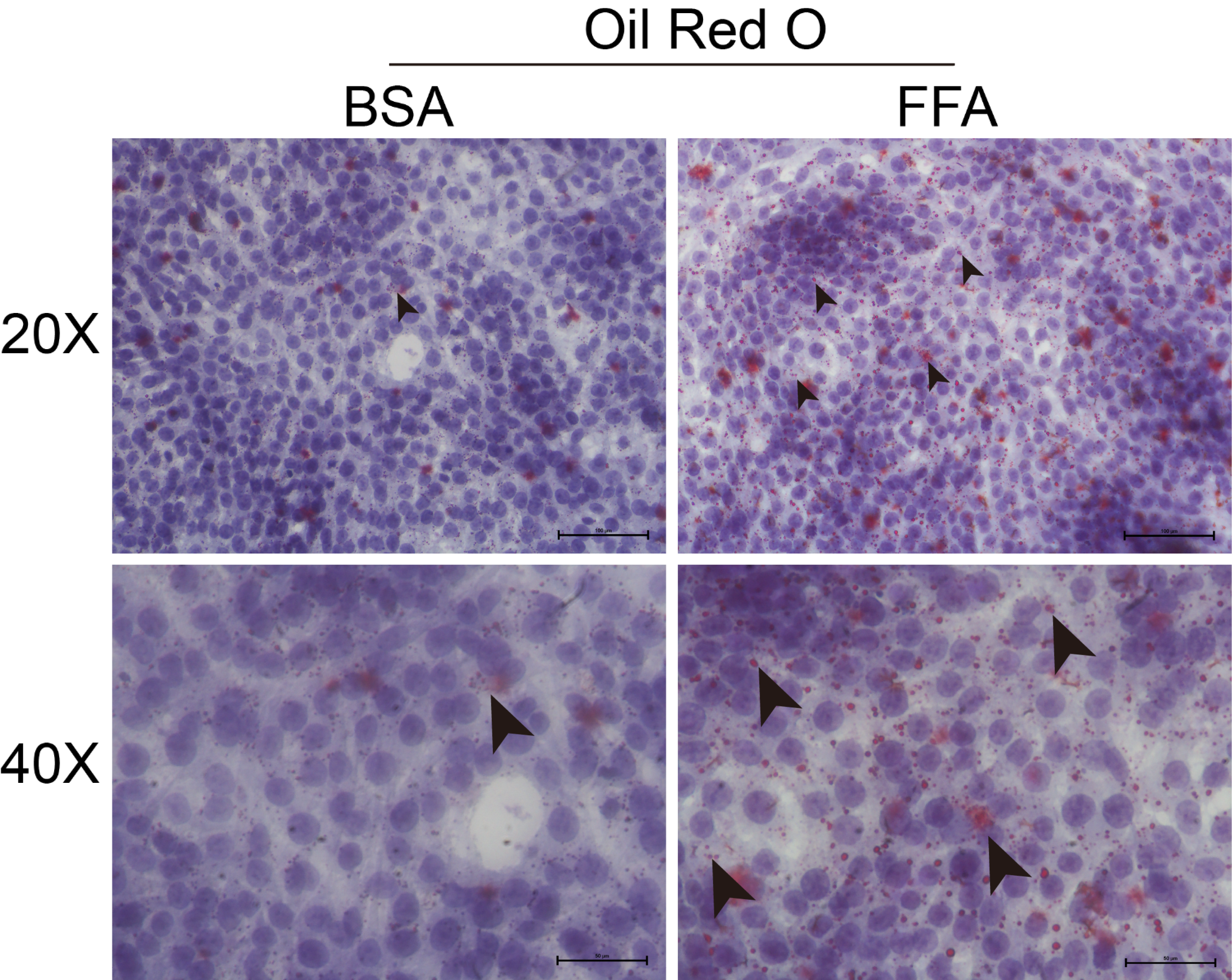

Supplement: Figure S1 — The oil red staining of HepG2, including BSA group (contrl) and FFA (hepatic steatosis model) group. [file peerj-10-13735-s001.png]

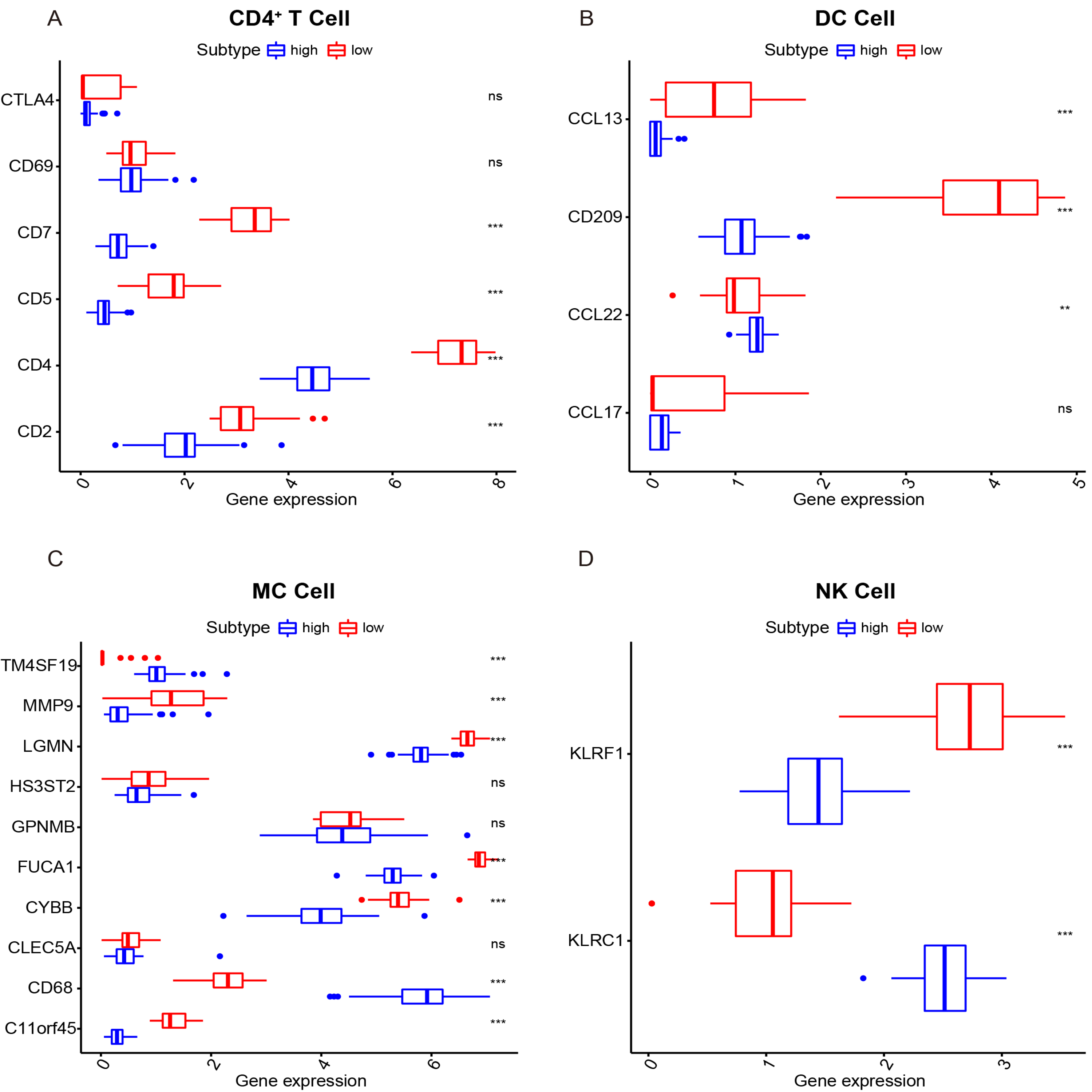

Supplement: Figure S2 — (A)-(D) Correlation analysis of GTF2I and immune cell surface markers. [file peerj-10-13735-s002.png]

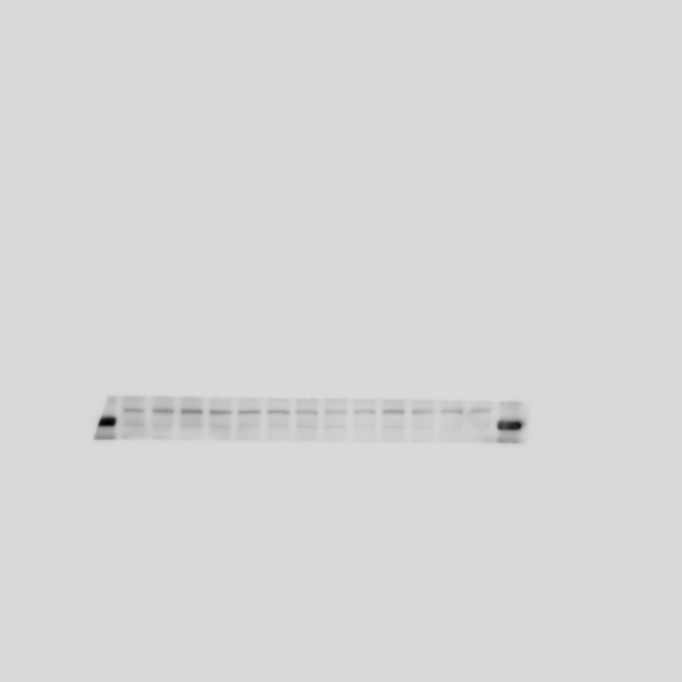

Supplement: Supplemental Information 2 [file peerj-10-13735-s004.zip › CHD2.tif]

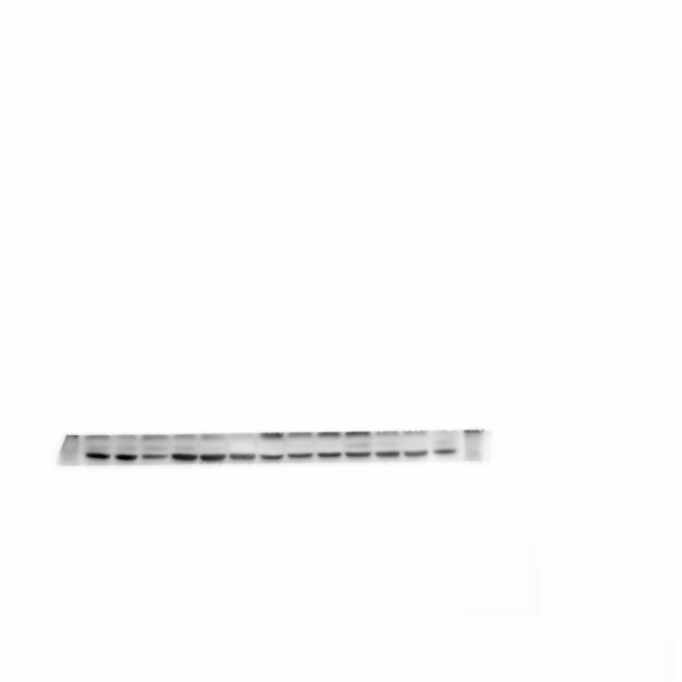

Supplement: Supplemental Information 2 [file peerj-10-13735-s004.zip › GAPDH.png]

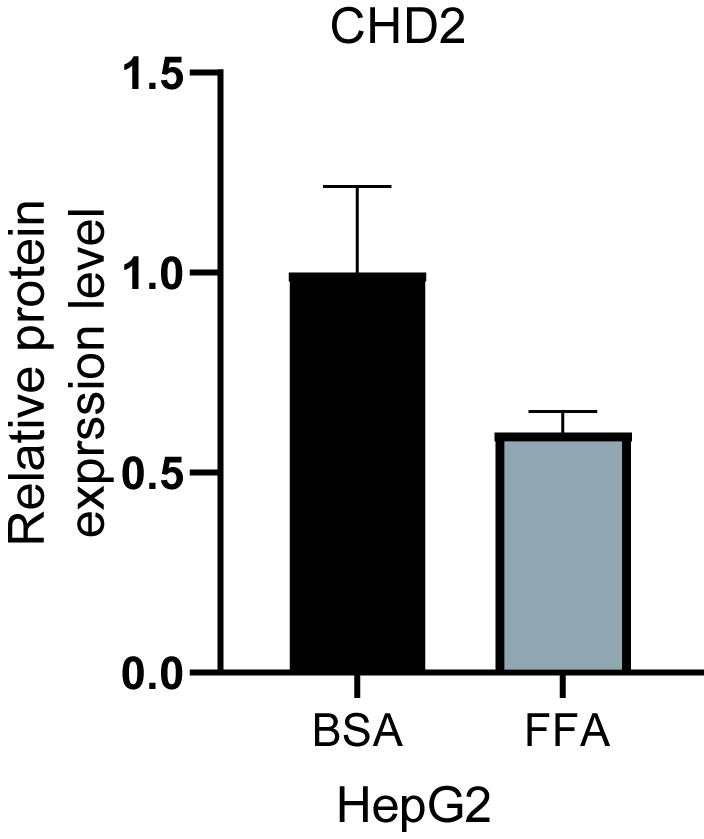

Supplement: Supplemental Information 2 [file peerj-10-13735-s004.zip › HepG2 CHD2 protein.tif]

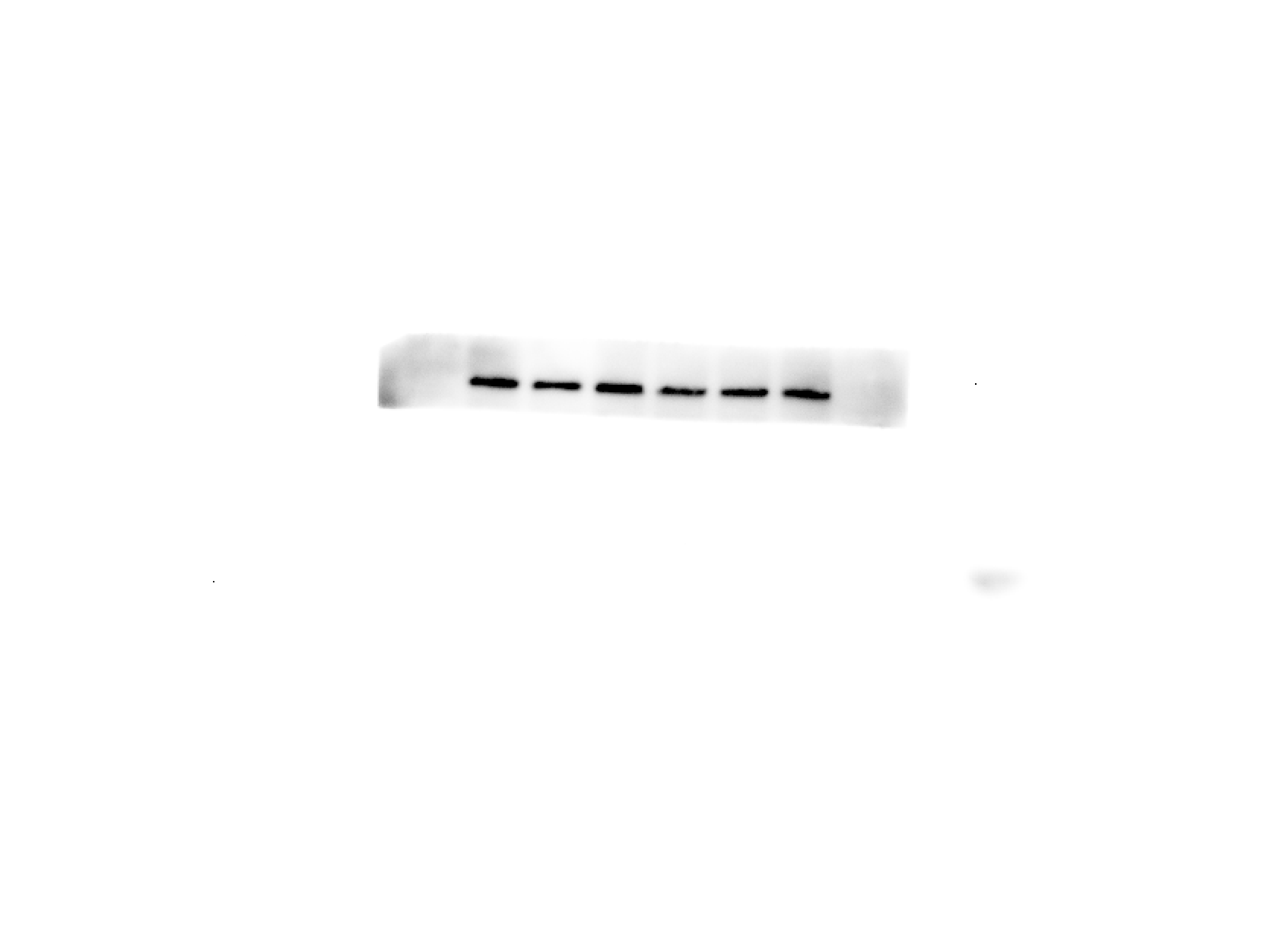

Supplement: Supplemental Information 2 [file peerj-10-13735-s004.zip › HepG2 CHD2.tif]

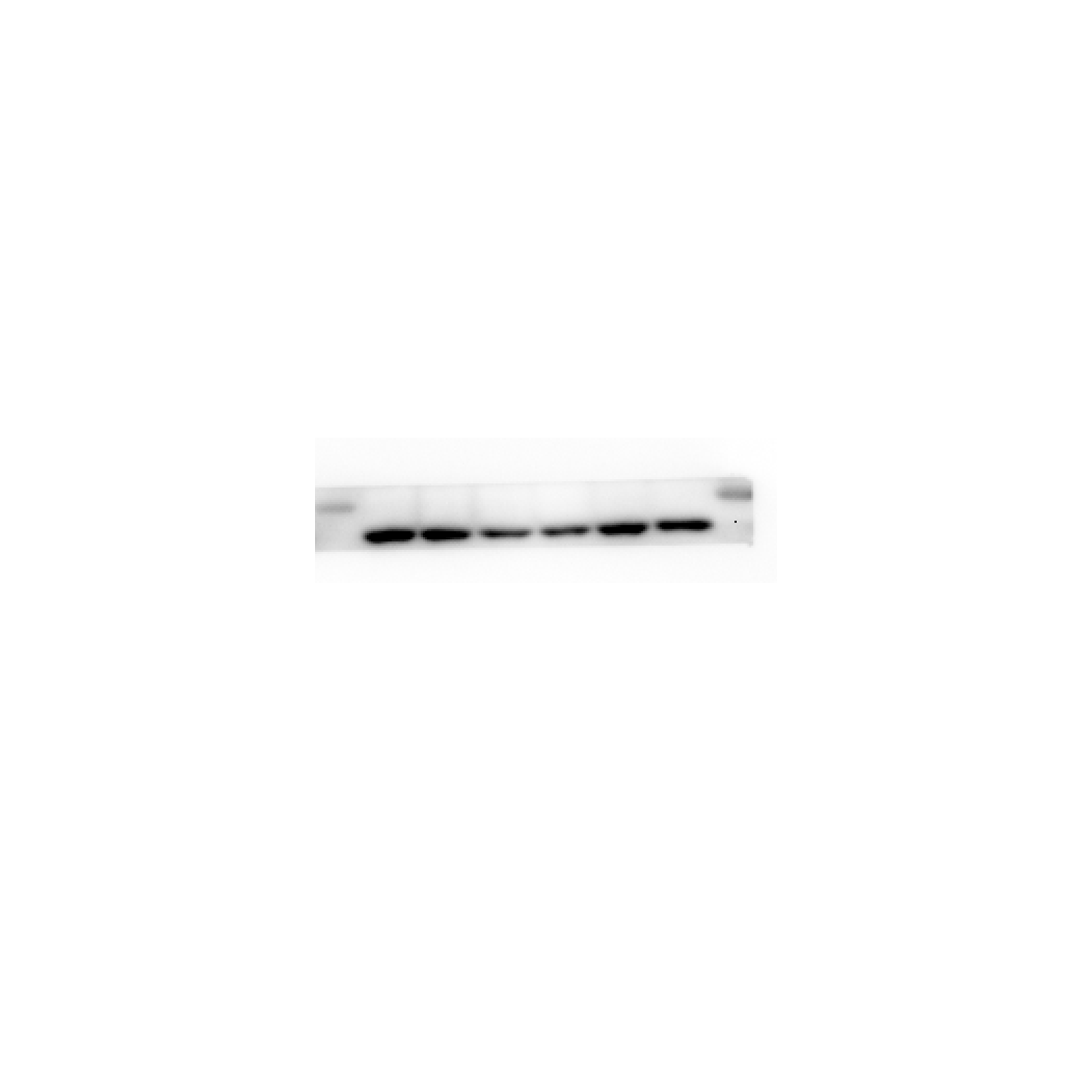

Supplement: Supplemental Information 2 [file peerj-10-13735-s004.zip › HepG2 GAPDH 1.tif]

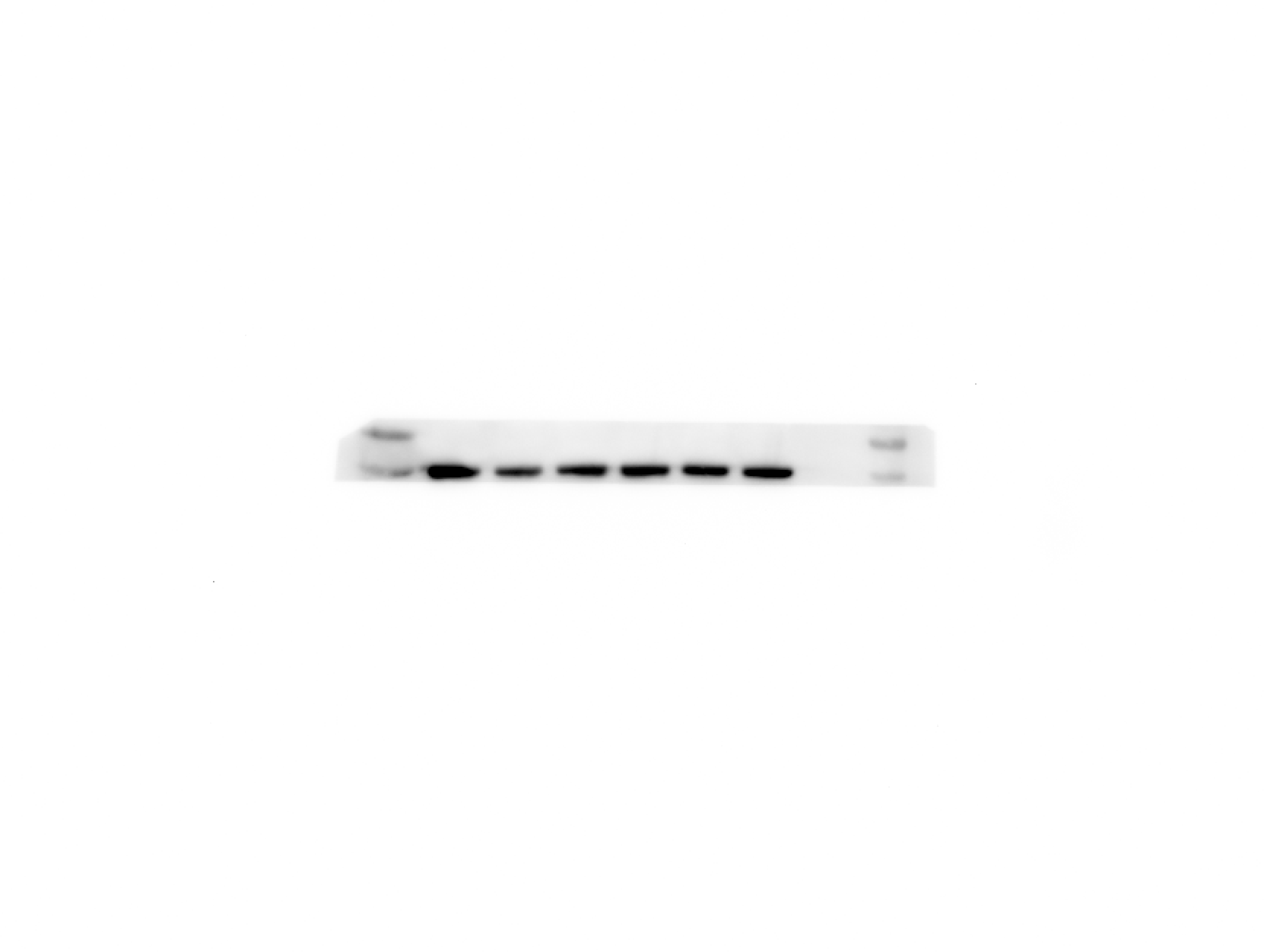

Supplement: Supplemental Information 2 [file peerj-10-13735-s004.zip › HepG2 GAPDH 2.tif]

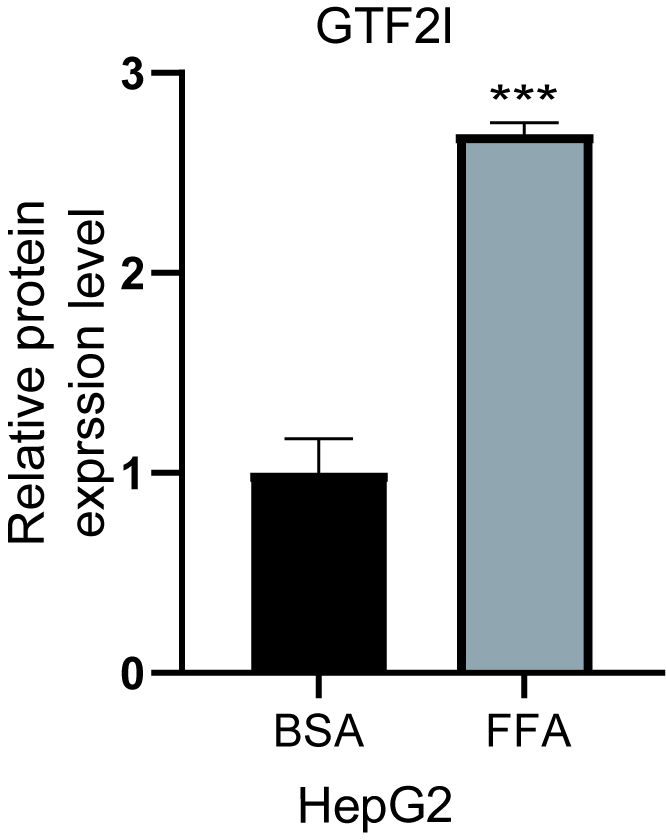

Supplement: Supplemental Information 2 [file peerj-10-13735-s004.zip › HepG2 GTF2I protein.tif]

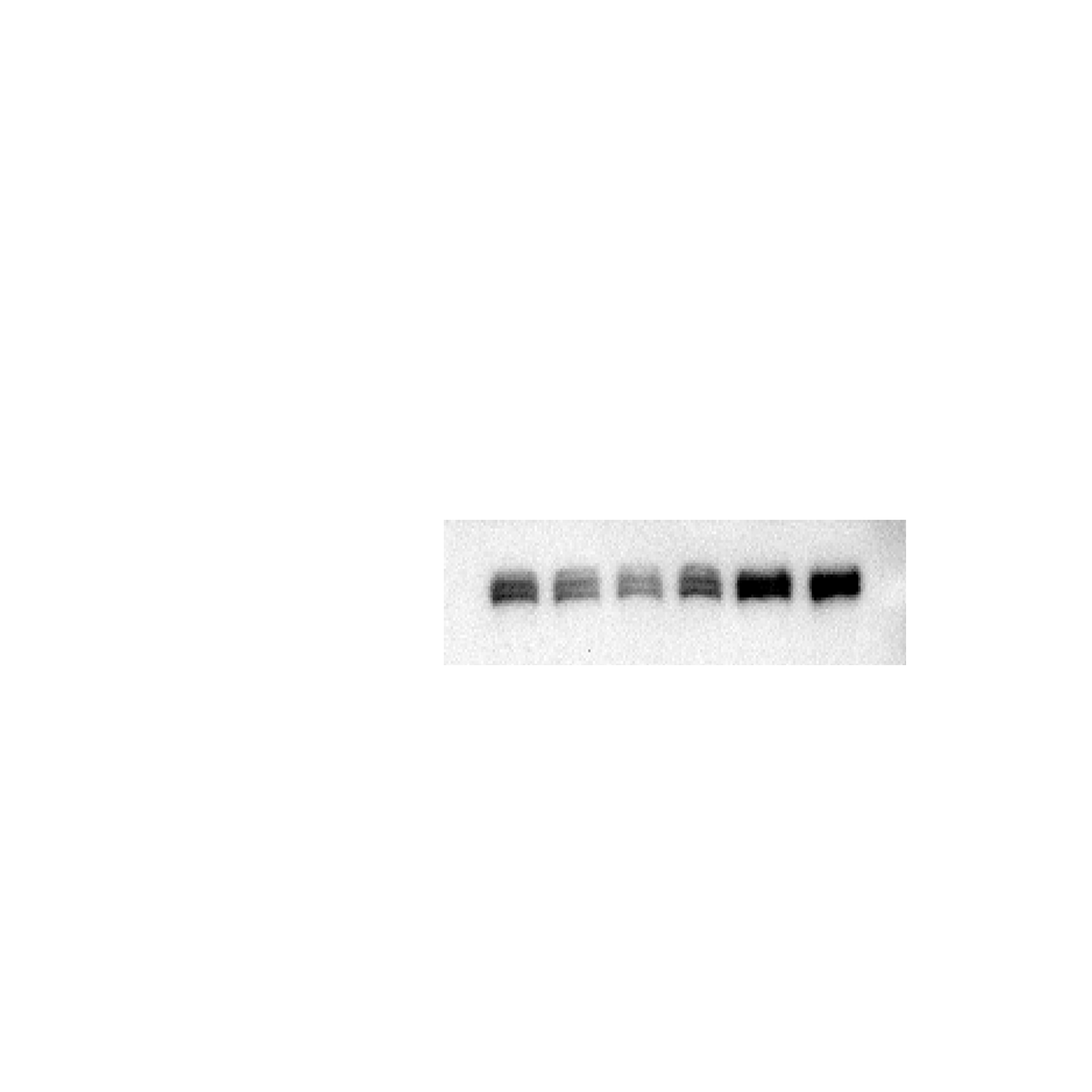

Supplement: Supplemental Information 2 [file peerj-10-13735-s004.zip › HepG2 GTF2I.tif]

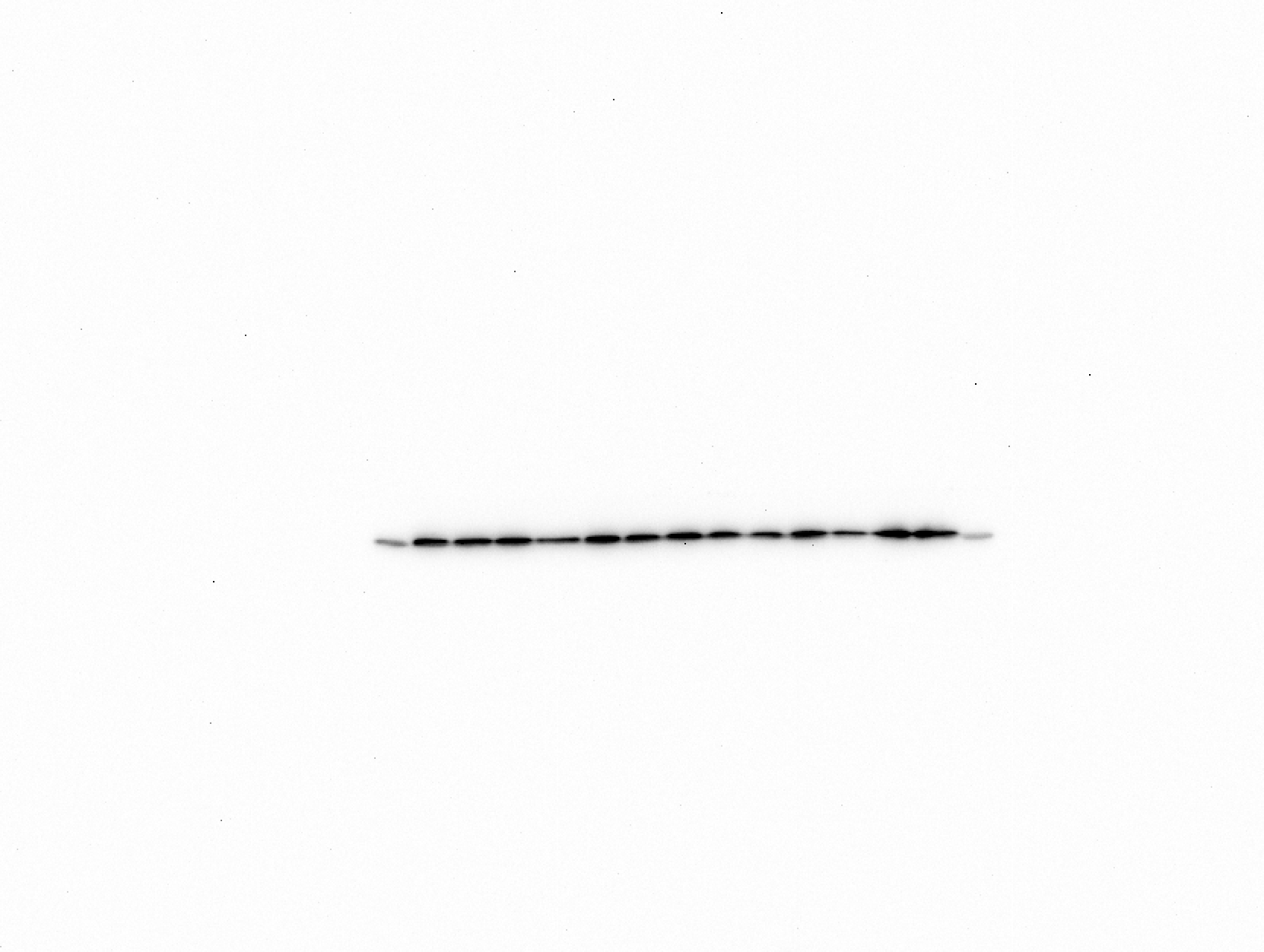

Supplement: Supplemental Information 2 [file peerj-10-13735-s004.zip › Human GAPDH.tif]

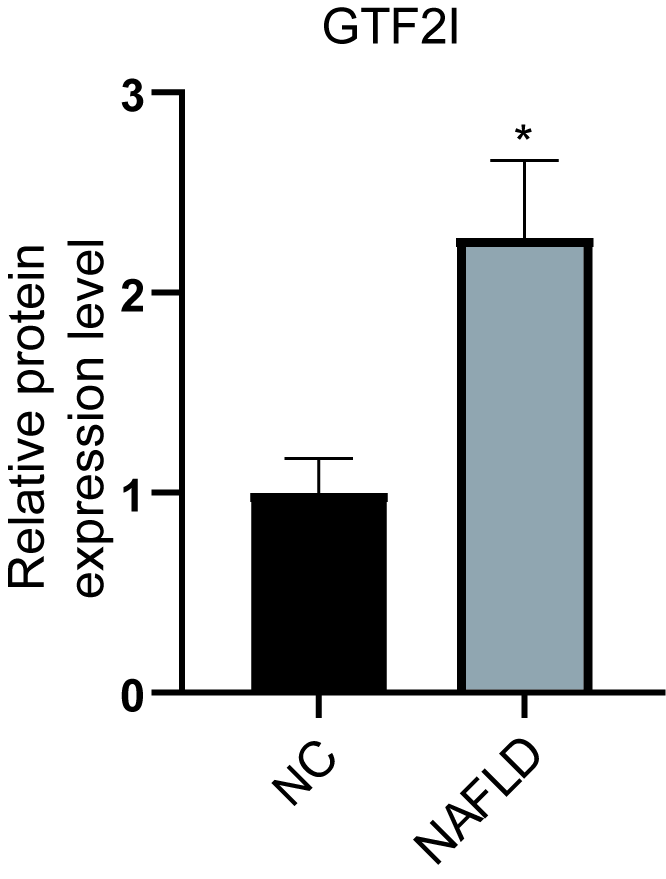

Supplement: Supplemental Information 2 [file peerj-10-13735-s004.zip › Human GTF2I protein.tif]

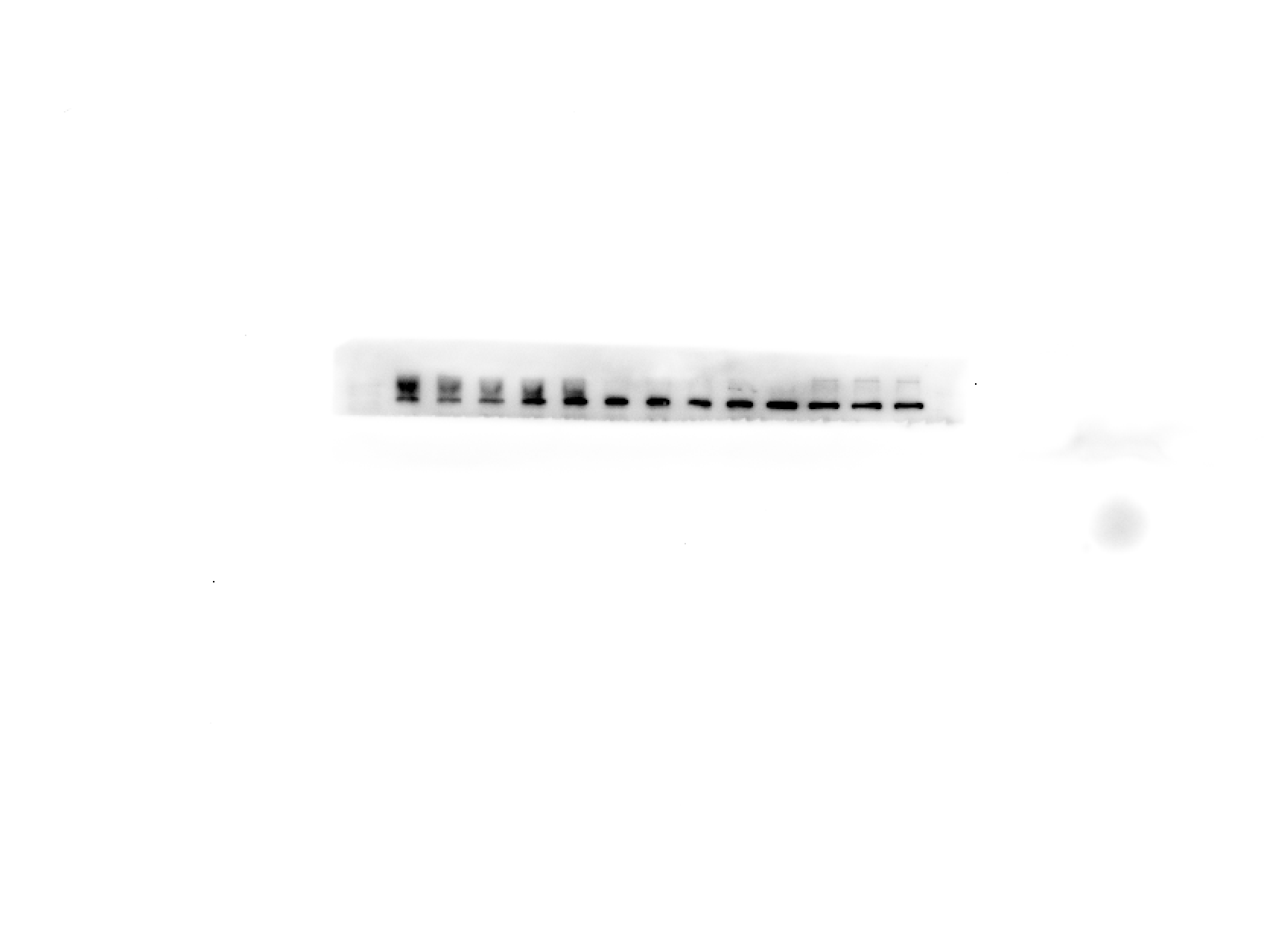

Supplement: Supplemental Information 2 [file peerj-10-13735-s004.zip › Human GTF2I.tif]
